# Crystal structure of (*Z*)-2-benzyl­idene-4-methyl-2*H*-benzo[*b*][1,4]thia­zin-3(4*H*)-one

**DOI:** 10.1107/S2056989015019295

**Published:** 2015-10-17

**Authors:** Mohamed Ellouz, Nada Kheira Sebbar, El Mokhtar Essassi, Mohamed Saadi, Lahcen El Ammari

**Affiliations:** a Laboratoire de Chimie Organique Hétérocyclique URAC 21, Pôle de Compétence Pharmacochimie, Faculté des Sciences, Université Mohammed V, Avenue Ibn Battouta, BP 1014, Rabat, Morocco; b Laboratoire de Chimie du Solide Appliquée, Faculté des Sciences, Université Mohammed V, Avenue Ibn Battouta, BP 1014, Rabat, Morocco

**Keywords:** crystal structure, thio­morpholin-3-one derivative, benzo­thia­zine derivative, hydrogen bonding

## Abstract

In the title compound, C_16_H_13_NOS, the 1,4-thia­zine ring displays a screw-boat conformation. The conformation about the ethene bond [1.344 (2) Å] is *Z*. The plane of the fused benzene ring makes a dihedral angle of 58.95 (9)° with the pendent phenyl ring, indicating a twisted conformation in the mol­ecule. In the crystal, mol­ecules are linked by pairs of C—H⋯O hydrogen bonds, forming inversion dimers.

## Related literature   

For background to the pharmacological activity and potential applications of benzo­thia­zines, see: Schiaffella *et al.* (2006 ▸); Gupta *et al.* (2009 ▸); Armenise *et al.* (2000 ▸); Bansode *et al.* (2009 ▸); Dixit *et al.* (2009 ▸); Dixit *et al.* (2008 ▸); Thomas *et al.* (2003 ▸). For medicinal applications; see: Warren *et al.* (1987 ▸); Armenise *et al.* (2012 ▸); Sabatini *et al.* (2008 ▸); Jacquot *et al.* (2001 ▸); Kalluraya *et al.* (2005 ▸); Munirajasekar *et al.* (2011 ▸). For similar compounds, see: Sebbar *et al.* (2014*a* ▸,*b* ▸); Zerzouf *et al.* (2001 ▸).
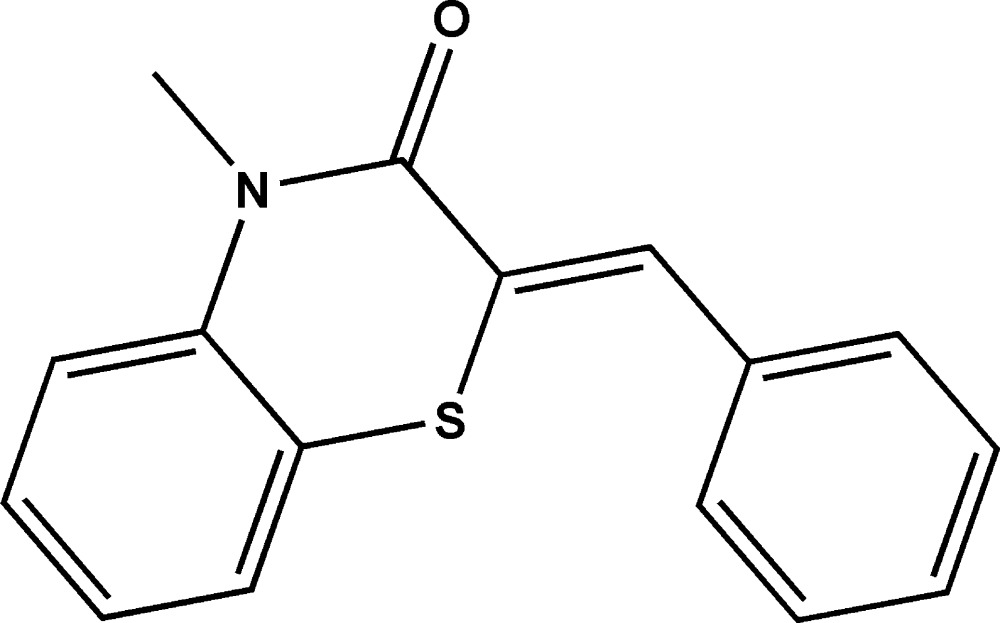


## Experimental   

### Crystal data  

C_16_H_13_NOS
*M* *_r_* = 267.33
Monoclinic, 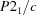

*a* = 9.1497 (3) Å
*b* = 14.7052 (5) Å
*c* = 10.0037 (3) Å
β = 97.051 (1)°
*V* = 1335.80 (7) Å^3^
*Z* = 4
Mo *K*α radiation
μ = 0.23 mm^−1^
*T* = 296 K
0.36 × 0.31 × 0.26 mm

### Data collection  

Bruker X8 APEX diffractometer
Absorption correction: multi-scan (*SADABS*; Bruker, 2009 ▸) *T*_min_ = 0.670, *T*_max_ = 0.746
25334 measured reflections
4082 independent reflections
3071 reflections with *I* > 2σ(*I*)
*R*_int_ = 0.031

### Refinement  

*R*[*F* ^2^ > 2σ(*F* ^2^)] = 0.048
*wR*(*F* ^2^) = 0.153
*S* = 1.12
4082 reflections
172 parameters
H-atom parameters constrained
Δρ_max_ = 0.37 e Å^−3^
Δρ_min_ = −0.21 e Å^−3^

### 

Data collection: *APEX2* (Bruker, 2009 ▸); cell refinement: *SAINT-Plus* (Bruker, 2009 ▸); data reduction: *SAINT-Plus*; program(s) used to solve structure: *SHELXS97* (Sheldrick, 2008 ▸); program(s) used to refine structure: *SHELXL97* (Sheldrick, 2008 ▸); molecular graphics: *ORTEP-3 for Windows* (Farrugia, 2012 ▸); software used to prepare material for publication: *PLATON* (Spek, 2009 ▸) and *publCIF* (Westrip, 2010 ▸).

## Supplementary Material

Crystal structure: contains datablock(s) I. DOI: 10.1107/S2056989015019295/tk5394sup1.cif

Structure factors: contains datablock(s) I. DOI: 10.1107/S2056989015019295/tk5394Isup2.hkl

Supporting information file. DOI: 10.1107/S2056989015019295/tk5394Isup3.cml

**Fig. 1.**
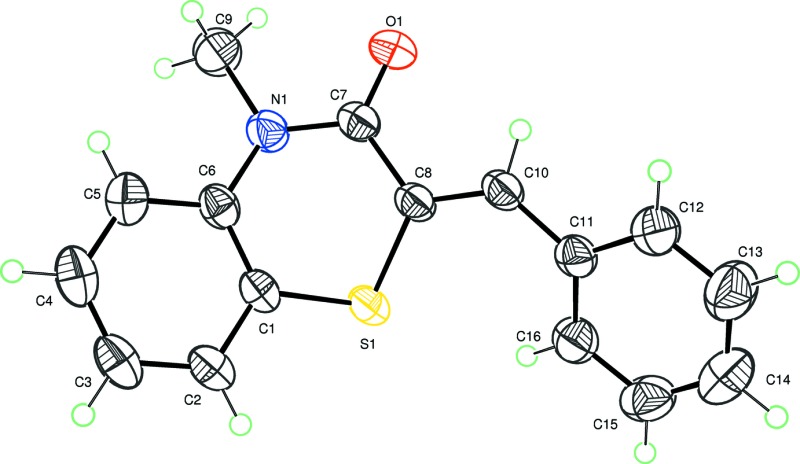
Molecular structure of the title compound with the atom-labeling scheme. Displacement ellipsoids are drawn at the 50% probability level. H atoms are represented as small circles.

. DOI: 10.1107/S2056989015019295/tk5394fig1.tif
Mol­ecular structure of the title compound with the atom-labeling scheme. Displacement ellipsoids are drawn at the 50% probability level. H atoms are represented as small circles.

**Fig. 2.**
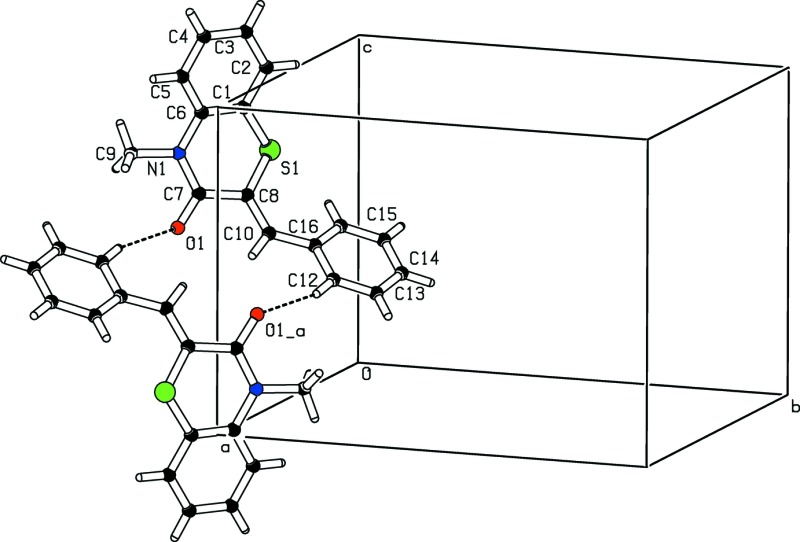
Supramolecular association in the title compound, showing inversion dimers of molecules linked through C12—H12···O1 hydrogen bond (dashed lines).

. DOI: 10.1107/S2056989015019295/tk5394fig2.tif
Supra­molecular association in the title compound, showing inversion dimers of mol­ecules linked through C12—H12⋯O1 hydrogen bond (dashed lines).

CCDC reference: 1430755

Additional supporting information:  crystallographic information; 3D view; checkCIF report

## Figures and Tables

**Table 1:** Hydrogen-bond geometry (, )

*D*H*A*	*D*H	H*A*	*D* *A*	*D*H*A*
C12H12O1^i^	0.93	2.50	3.404(2)	164

Symmetry code: (i) 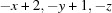.

## supplementary crystallographic information

### S1. Comment

Over the years, 4*H*-1,4-benzothiazines have constituted an important class of heterocycles which, even when part of a complex molecule, possess a wide spectrum of biological activities (Schiaffella *et al.*, 2006; Gupta *et al.*, 2009; Armenise *et al.*, 2000). Due to the presence of a fold along the nitrogen—sulfur axis, the biological activity of some 1,4-benzothiazines is similar to that of phenothiazines, featuring the same structural specificity (Bansode *et al.*, 2009; Dixit *et al.*, 2009; Dixit *et al.*, 2008; Thomas *et al.*, 2003). Generally, benzothiazine and derivatives have found widespread application as analgesic (Warren *et al.*, 1987), antibacterial (Armenise *et al.*, 2012; Sabatini *et al.*, 2008), anticancer (Jacquot *et al.*, 2001), anticonvulsant (Kalluraya *et al.*, 2005) and anthelmintic (Munirajasekar *et al.*, 2011) agents. As a continuation of our research work devoted to the development of N-substituted benzothiazine and evaluating their potential pharmacological activities, we have checked the action of iodomethane towards (*E*)-2-benzylidene-2*H*-benzo[*b*][1,4]thiazin-3(4*H*)-one under phase-transfer catalysis conditions using tetra n-butylammonium bromide (TBAB) as catalyst and potassium carbonate as base (Sebbar *et al.*, 2014*a*, Sebbar *et al.*, 2014*b*; Zerzouf *et al.*, 2001). This led to the characterization of the title compound, Scheme 1.

The molecule of the title compound is build up from two fused six-membered rings linked as shown in Fig.1. The dihedral angle between the (C1 to C6) and (C11 to C16) benzene rings is 58.95 (9)°.

In the crystal, the molecules are linked together by a hydrogen bond (C12–H12···O1) in a way to build a dimer, as shown in Fig. 2 and Table 1.

### S2. Experimental

To a solution of 2-(benzylidene)-3,4-dihydro-2*H*-1,4-benzothiazin-3-one (0.5 g, 2 mmol), potassium carbonate (0.55 g, 4 mmol) and tetra *n*-butyl ammonium bromide (0.064 g, 0.2 mmol) in DMF (15 ml) was added iodomethane (0.25 ml, 4 mmol). Stirring was continued at room temperature for 12 h. The mixture was filtered and the solvent removed. The residue was extracted with water. The organic compound was chromatographed on a column of silica gel with ethyl acetate-hexane (9/1) as eluent. Brown crystals were isolated when the solvent was allowed to evaporate (yield: 53%; m.p. = 342 K).

### S3. Refinement

The H atoms were located in a difference map and treated as riding with C—H = 0.93 Å (aromatic) and C—H = 0.96 Å (methyl), and with *U*_iso_(H) = 1.2 *U*_eq_ (aromatic) and *U*_iso_(H) = 1.5 *U*_eq_ (methyl).

### Crystal data

**Table:**

C_16_H_13_NOS	*D*_x_ = 1.329 Mg m^−^^3^
*M**_r_* = 267.33	Melting point: 342 K
Monoclinic, *P*2_1_/*c*	Mo *K*α radiation, λ = 0.71073 Å
*a* = 9.1497 (3) Å	Cell parameters from 4082 reflections
*b* = 14.7052 (5) Å	θ = 2.2–30.5°
*c* = 10.0037 (3) Å	µ = 0.23 mm^−^^1^
β = 97.051 (1)°	*T* = 296 K
*V* = 1335.80 (7) Å^3^	Prism, brown
*Z* = 4	0.36 × 0.31 × 0.26 mm
*F*(000) = 560	

### Data collection

**Table:**

Bruker X8 APEX diffractometer	4082 independent reflections
Radiation source: fine-focus sealed tube	3071 reflections with *I* > 2σ(*I*)
Graphite monochromator	*R*_int_ = 0.031
φ and ω scans	θ_max_ = 30.5°, θ_min_ = 2.2°
Absorption correction: multi-scan (*SADABS*; Bruker, 2009)	*h* = −7→13
*T*_min_ = 0.670, *T*_max_ = 0.746	*k* = −20→21
25334 measured reflections	*l* = −14→14

### Refinement

**Table:**

Refinement on *F*^2^	0 restraints
Least-squares matrix: full	Hydrogen site location: inferred from neighbouring sites
*R*[*F*^2^ > 2σ(*F*^2^)] = 0.048	H-atom parameters constrained
*wR*(*F*^2^) = 0.153	*w* = 1/[σ^2^(*F*_o_^2^) + (0.0757*P*)^2^ + 0.2379*P*] where *P* = (*F*_o_^2^ + 2*F*_c_^2^)/3
*S* = 1.12	(Δ/σ)_max_ < 0.001
4082 reflections	Δρ_max_ = 0.37 e Å^−^^3^
172 parameters	Δρ_min_ = −0.21 e Å^−^^3^

### Special details

**Table:**

Geometry. All e.s.d.'s (except the e.s.d. in the dihedral angle between two l.s. planes) are estimated using the full covariance matrix. The cell e.s.d.'s are taken into account individually in the estimation of e.s.d.'s in distances, angles and torsion angles; correlations between e.s.d.'s in cell parameters are only used when they are defined by crystal symmetry. An approximate (isotropic) treatment of cell e.s.d.'s is used for estimating e.s.d.'s involving l.s. planes.

### Fractional atomic coordinates and isotropic or equivalent isotropic displacement parameters (Å^2^)

**Table:**

	*x*	*y*	*z*	*U*_iso_*/*U*_eq_	
C1	0.73318 (17)	0.52736 (10)	0.43834 (14)	0.0426 (3)	
C2	0.6952 (2)	0.48778 (12)	0.55605 (16)	0.0513 (4)	
H2	0.6186	0.4459	0.5516	0.062*	
C3	0.7715 (2)	0.51089 (13)	0.67964 (16)	0.0579 (5)	
H3	0.7445	0.4862	0.7586	0.070*	
C4	0.8872 (2)	0.57054 (13)	0.68451 (17)	0.0596 (4)	
H4	0.9383	0.5863	0.7675	0.071*	
C5	0.9292 (2)	0.60761 (12)	0.56848 (17)	0.0549 (4)	
H5	1.0100	0.6464	0.5737	0.066*	
C6	0.85101 (17)	0.58725 (10)	0.44272 (14)	0.0425 (3)	
C7	0.86549 (16)	0.58789 (10)	0.19776 (14)	0.0435 (3)	
C8	0.75763 (16)	0.51143 (10)	0.17601 (14)	0.0408 (3)	
C9	0.9905 (3)	0.70539 (13)	0.3367 (2)	0.0689 (5)	
H9A	1.0003	0.7272	0.4278	0.103*	
H9B	1.0853	0.6874	0.3142	0.103*	
H9C	0.9512	0.7528	0.2768	0.103*	
C10	0.75953 (16)	0.45936 (11)	0.06583 (14)	0.0430 (3)	
H10	0.8336	0.4735	0.0133	0.052*	
C11	0.66396 (17)	0.38420 (10)	0.01526 (14)	0.0432 (3)	
C12	0.7201 (2)	0.32215 (12)	−0.07117 (18)	0.0563 (4)	
H12	0.8150	0.3299	−0.0935	0.068*	
C13	0.6368 (3)	0.24967 (15)	−0.1236 (3)	0.0770 (6)	
H13	0.6763	0.2088	−0.1804	0.092*	
C14	0.4954 (3)	0.23710 (15)	−0.0928 (2)	0.0744 (6)	
H14	0.4397	0.1879	−0.1283	0.089*	
C15	0.4378 (2)	0.29778 (15)	−0.0094 (2)	0.0663 (5)	
H15	0.3427	0.2893	0.0121	0.080*	
C16	0.51940 (18)	0.37157 (13)	0.04339 (17)	0.0550 (4)	
H16	0.4778	0.4130	0.0979	0.066*	
N1	0.89082 (15)	0.62709 (9)	0.32356 (13)	0.0466 (3)	
O1	0.92759 (14)	0.61644 (9)	0.10488 (12)	0.0591 (3)	
S1	0.62428 (4)	0.50183 (3)	0.28662 (4)	0.05206 (15)	

### Atomic displacement parameters (Å^2^)

**Table:**

	*U*^11^	*U*^22^	*U*^33^	*U*^12^	*U*^13^	*U*^23^
C1	0.0506 (8)	0.0451 (7)	0.0335 (6)	0.0048 (6)	0.0115 (6)	0.0010 (5)
C2	0.0614 (10)	0.0554 (9)	0.0395 (7)	0.0038 (7)	0.0162 (7)	0.0053 (6)
C3	0.0759 (12)	0.0629 (10)	0.0359 (8)	0.0138 (8)	0.0103 (8)	0.0093 (7)
C4	0.0735 (11)	0.0637 (10)	0.0390 (7)	0.0134 (9)	−0.0027 (7)	−0.0030 (7)
C5	0.0626 (10)	0.0528 (9)	0.0479 (8)	0.0018 (7)	0.0014 (7)	−0.0070 (7)
C6	0.0537 (8)	0.0380 (7)	0.0367 (7)	0.0064 (6)	0.0090 (6)	−0.0005 (5)
C7	0.0451 (7)	0.0476 (8)	0.0390 (7)	−0.0009 (6)	0.0101 (6)	0.0039 (6)
C8	0.0405 (7)	0.0504 (8)	0.0322 (6)	−0.0003 (6)	0.0079 (5)	0.0045 (5)
C9	0.0953 (15)	0.0483 (9)	0.0663 (11)	−0.0214 (9)	0.0228 (10)	−0.0069 (8)
C10	0.0425 (7)	0.0545 (8)	0.0327 (6)	−0.0003 (6)	0.0077 (5)	0.0038 (6)
C11	0.0459 (7)	0.0500 (8)	0.0328 (6)	−0.0006 (6)	0.0019 (5)	0.0059 (5)
C12	0.0566 (9)	0.0543 (9)	0.0589 (10)	−0.0009 (7)	0.0107 (8)	−0.0052 (7)
C13	0.0818 (14)	0.0585 (11)	0.0914 (16)	−0.0055 (10)	0.0138 (12)	−0.0195 (10)
C14	0.0754 (13)	0.0584 (11)	0.0862 (14)	−0.0158 (10)	−0.0033 (11)	−0.0036 (10)
C15	0.0503 (9)	0.0789 (13)	0.0677 (11)	−0.0139 (9)	−0.0012 (8)	0.0097 (10)
C16	0.0465 (8)	0.0704 (11)	0.0474 (8)	−0.0025 (7)	0.0033 (6)	−0.0006 (8)
N1	0.0583 (8)	0.0404 (6)	0.0429 (6)	−0.0045 (5)	0.0130 (5)	0.0001 (5)
O1	0.0633 (7)	0.0710 (8)	0.0461 (6)	−0.0177 (6)	0.0191 (5)	0.0037 (5)
S1	0.0473 (2)	0.0749 (3)	0.0361 (2)	−0.01066 (18)	0.01376 (16)	−0.00324 (16)

### Geometric parameters (Å, º)

**Table:**

C1—C6	1.389 (2)	C9—N1	1.465 (2)
C1—C2	1.395 (2)	C9—H9A	0.9600
C1—S1	1.7510 (15)	C9—H9B	0.9600
C2—C3	1.386 (3)	C9—H9C	0.9600
C2—H2	0.9300	C10—C11	1.461 (2)
C3—C4	1.371 (3)	C10—H10	0.9300
C3—H3	0.9300	C11—C12	1.398 (2)
C4—C5	1.379 (3)	C11—C16	1.398 (2)
C4—H4	0.9300	C12—C13	1.376 (3)
C5—C6	1.401 (2)	C12—H12	0.9300
C5—H5	0.9300	C13—C14	1.379 (3)
C6—N1	1.4154 (19)	C13—H13	0.9300
C7—O1	1.2215 (18)	C14—C15	1.371 (3)
C7—N1	1.3777 (19)	C14—H14	0.9300
C7—C8	1.494 (2)	C15—C16	1.385 (3)
C8—C10	1.344 (2)	C15—H15	0.9300
C8—S1	1.7505 (15)	C16—H16	0.9300
			
C6—C1—C2	120.73 (14)	H9A—C9—H9C	109.5
C6—C1—S1	121.35 (11)	H9B—C9—H9C	109.5
C2—C1—S1	117.89 (13)	C8—C10—C11	130.37 (14)
C3—C2—C1	120.00 (17)	C8—C10—H10	114.8
C3—C2—H2	120.0	C11—C10—H10	114.8
C1—C2—H2	120.0	C12—C11—C16	117.82 (15)
C4—C3—C2	119.42 (16)	C12—C11—C10	117.27 (14)
C4—C3—H3	120.3	C16—C11—C10	124.87 (15)
C2—C3—H3	120.3	C13—C12—C11	120.86 (18)
C3—C4—C5	121.10 (16)	C13—C12—H12	119.6
C3—C4—H4	119.5	C11—C12—H12	119.6
C5—C4—H4	119.5	C12—C13—C14	120.6 (2)
C4—C5—C6	120.48 (17)	C12—C13—H13	119.7
C4—C5—H5	119.8	C14—C13—H13	119.7
C6—C5—H5	119.8	C15—C14—C13	119.39 (19)
C1—C6—C5	118.21 (14)	C15—C14—H14	120.3
C1—C6—N1	121.00 (13)	C13—C14—H14	120.3
C5—C6—N1	120.79 (15)	C14—C15—C16	120.82 (19)
O1—C7—N1	120.60 (14)	C14—C15—H15	119.6
O1—C7—C8	120.56 (14)	C16—C15—H15	119.6
N1—C7—C8	118.81 (12)	C15—C16—C11	120.45 (18)
C10—C8—C7	118.25 (13)	C15—C16—H16	119.8
C10—C8—S1	123.55 (12)	C11—C16—H16	119.8
C7—C8—S1	117.89 (11)	C7—N1—C6	124.35 (12)
N1—C9—H9A	109.5	C7—N1—C9	116.37 (13)
N1—C9—H9B	109.5	C6—N1—C9	118.12 (13)
H9A—C9—H9B	109.5	C8—S1—C1	99.44 (7)
N1—C9—H9C	109.5		

### Hydrogen-bond geometry (Å, º)

**Table:**

*D*—H···*A*	*D*—H	H···*A*	*D*···*A*	*D*—H···*A*
C12—H12···O1^i^	0.93	2.50	3.404 (2)	164

Symmetry code: (i) −*x*+2, −*y*+1, −*z*.
