# Supplementary figures and images for: Crystal structure of (Z)-2-benzyl­idene-4-methyl-2H-benzo[b][1,4]thia­zin-3(4H)-one
Source: Acta Crystallogr E Crystallogr Commun. 2015 Oct 17;71(Pt 11):o862–3. doi: 10.1107/S2056989015019295 (PMC4645059; doi:10.1107/S2056989015019295)

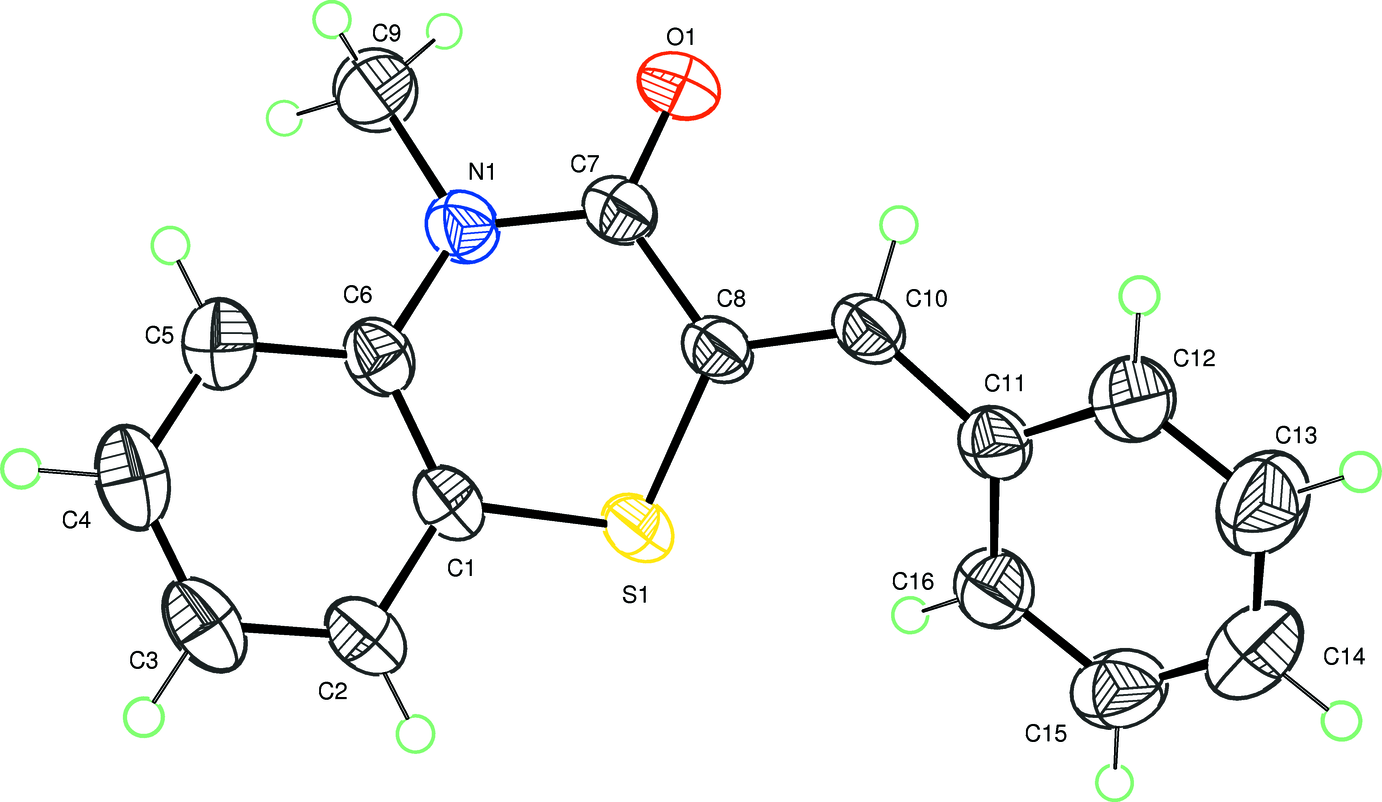

Supplement: Supplementary file 4 [file e-71-0o862-fig1.tif]

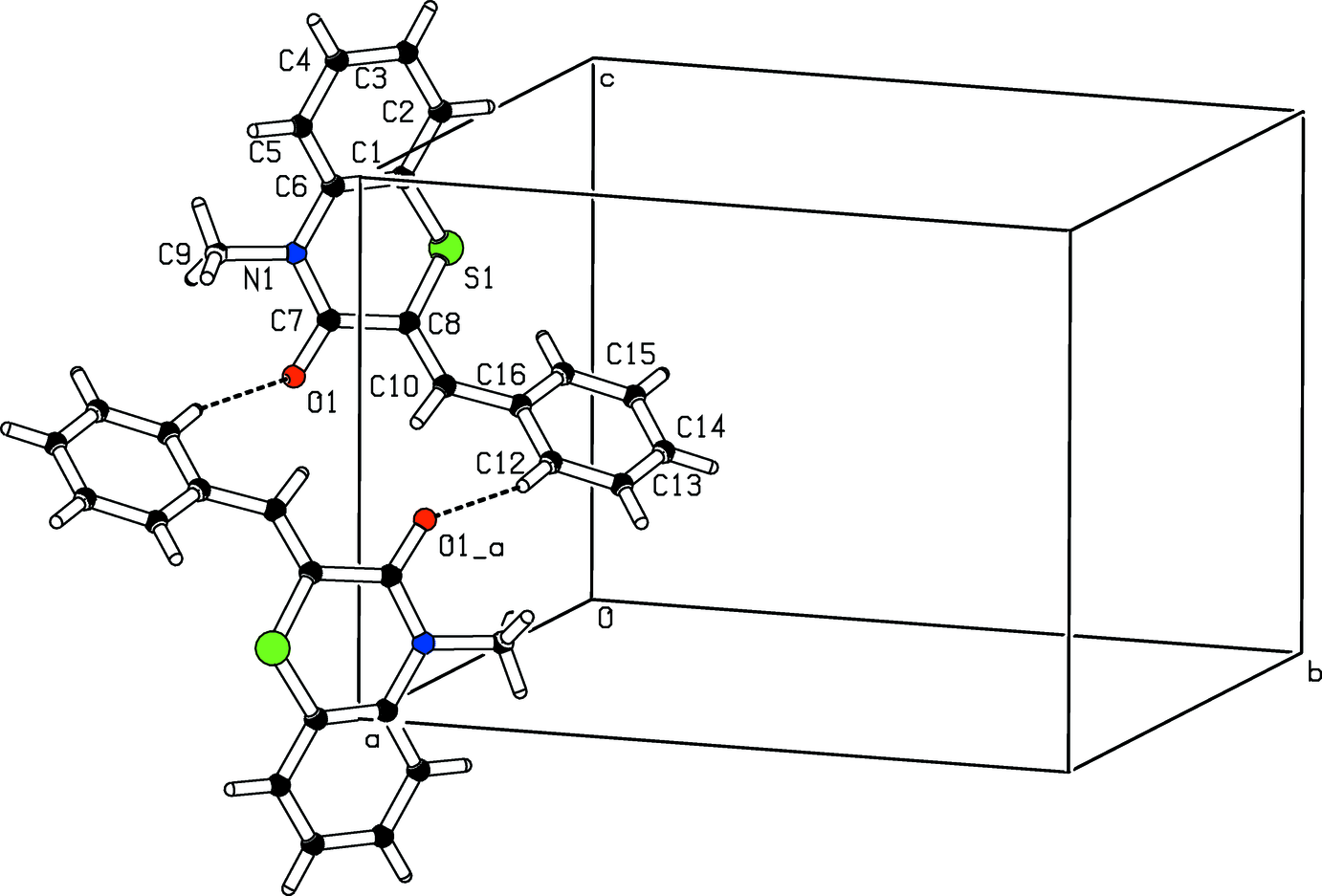

Supplement: Supplementary file 5 [file e-71-0o862-fig2.tif]
